# The relative proportion of comorbidities among rhinitis and rhinosinusitis patients and their impact on visit burden

**DOI:** 10.1002/clt2.12181

**Published:** 2022-07-21

**Authors:** Mikko Nuutinen, Annina Lyly, Paula Virkkula, Maija Hytönen, Elmo Saarentaus, Antti Mäkitie, Aarno Palotie, Paulus Torkki, Jari Haukka, Sanna Toppila‐Salmi

**Affiliations:** ^1^ Haartman Institute University of Helsinki Helsinki Finland; ^2^ Skin and Allergy Hospital University of Helsinki and Helsinki University Hospital Helsinki Finland; ^3^ Department of Otorhinolaryngology–Head and Neck Surgery Helsinki University Hospital and University of Helsinki Helsinki Finland; ^4^ HiLIFE Institute for Molecular Medicine Finland (FIMM) University of Helsinki Helsinki Finland; ^5^ Analytic and Translational Genetics Unit Massachusetts General Hospital Boston Massachusetts USA; ^6^ Program in Medical and Population Genetics Broad Institute of Harvard and MIT Cambridge Massachusetts USA; ^7^ Stanley Center for Psychiatric Research Broad Institute of Harvard and MIT Cambridge Massachusetts USA; ^8^ Department of Public Health University of Helsinki Helsinki Finland; ^9^ Department of Pulmonary Medicine Heart and Lung Center Helsinki University Hospital and University of Helsinki Helsinki Finland

**Keywords:** allergy, asthma, chronic rhinosinusitis, non‐steroidal anti‐inflammatory drug exacerbated respiratory disease, rhinitis

## Abstract

**Background:**

The aim was to evaluate the relative proportion of Non‐steroidal anti‐inflammatory drug exacerbated respiratory disease (NERD) and other comorbidities, and their impact on the burden of outpatient visits due to allergic rhinitis (AR), non‐allergic rhinitis (NAR), acute rhinosinusitis (ARS), and chronic rhinosinusitis with nasal polyps (CRSwNP) and without (CRSsNP).

**Methods:**

We used hospital registry data of a random sample of 5080 rhinitis/rhinosinusitis patients diagnosed during 2005–2019. International Statistical Classification of Diseases and Related Health Problems (ICD10) diagnoses, visits, and other factors were collected from electronic health records by using information extraction and data processing methods. Cox's proportional hazards model was used for modeling the time to the next outpatient visit.

**Results:**

The mean (±standard deviation) age of the population was 33.6 (±20.7) years and 56.1% were female. The relative proportion of AR, NAR, ARS, CRSsNP and CRSwNP, were 33.5%, 27.5%, 27.2%, 20.7%, and 10.9%, respectively. The most common other comorbidities were asthma (44.4%), other chronic respiratory diseases (38.5%), musculoskeletal diseases (38.4%), and cardiovascular diseases (35.7%). Non‐steroidal anti‐inflammatory drug exacerbated respiratory disease existed in 3.9% of all patients, and 17.7% of the CRSwNP group. The relative proportion of subjects having 1, 2, 3 and ≥ 4 other diseases were 18.0%, 17.6%, 17.0%, 37.0%, respectively. All diseases except AR, ARS, and mouth breathing, were associated with a high frequency of outpatient visits.

**Conclusions:**

Our results revealed a high relative proportion of NERD and other comorbidities, which affect the burden of outpatient visits and hence confirm the socioeconomic impact of upper airway diseases.

## 1 BACKGROUND

Chronic inflammatory sinonasal diseases include rhinosinusitis (CRS), allergic rhinitis (AR), non‐allergic rhinitis (NAR), and non‐steroidal anti‐inflammatory drug‐exacerbated respiratory disease (NERD). They carry a significant health and economic burden.^1^, ^2^, ^3^, ^4^, ^5^

The prevalence of AR is up to 50%,^2^ and that of NAR is 6%–25%.^3^, ^6^ The prevalence of CRS without nasal polyps (CRSsNP) is about 11% in the general population^1^ and with nasal polyps (CRSwNP) about 1%–4%.^4^ Risk factors of these diseases include asthma, other allergic diseases, NERD, and smoking, in addition to genetic predisposition and host‐environmental (‐microbial) interactions. About 10% have severe disease, of which 70% have type 2 (eosinophilic) inflammation, CRSwNP, asthma/allergic multi‐morbidity, and/or NERD, whereas the remaining part of the uncontrolled cases has variable risk factors.^7^, ^8^, ^9^ The proportion of NERD has shown to be about 16% among hospital CRSwNP patients.^10^

Multiple chronic conditions have been shown to increase the frequency of physician visits.^11^ We have previously shown that patients with at least one chronic disease have an increased risk of severe asthma.^9^ We are not aware of previous literature on the overlap of diagnoses, comorbidities, and burden of outpatient visits due to rhinitis/rhinosinusitis.

This study was carried out to evaluate the relative proportion of NERD and other comorbidities and their impact on the burden of outpatient visits due to AR/NAR/ARS/CRS. Although inflammatory upper airway diseases have been shown to have a significant socio‐economic impact, their outpatient visit burden has been scarcely studied.

## 2 MATERIALS AND METHODS

### 2.1 Patients

This retrospective registry‐based follow‐up study on rhinitis or rhinosinusitis patients was carried out at the Departments of Allergy and Otorhinolaryngology–Head and Neck Surgery, in the Hospital District of Helsinki and Uusimaa (HUS), Finland. The study (nro 31/13/03/00/2015) was approved by the research committee and institutional research permission was granted.

The study population comprised a random sample of patients (*n* = 5080) with the diagnosis of J30., J31., J32., J33. or J01 registered at outpatient visits. The longitudinal data of random patient samples (including patients of any age) from the electronic health records (EHR) were collected from the years 2005, 2007, 2009, 2011, and 2013, with equally sized samples each month and year. The last follow‐up data collection time point for all patients was September 30, 2019.

The patient variables for the study were collected and processed both from the structured and coded EHR data (c.f. Visits, procedure, and diagnosis codes) and free text from the hospital charts.

### 2.2 The collected variables

- Personal characteristics (*n* = 2): gender, age
- Diseases of interest (*n* = 4): NAR (J31.), AR (J30.), CRS (J32., J33.), ARS (J01.)
- Phenotypes of interest (*n* = 2): CRSwNP (J33.), CRSsNP (J32. + no J33. + no existing EHR of nasal polyps), NERD (keyword search, see Table E1 in the [Additional file 1])
- Acute rhinosinusitis (*n* = 4)
  ‐ ARS (acute purulent rhinosinusitis J01.; ≤3 visits J01, so that the time between visits 1. And 2. Is ≤ 90 days, there was no diagnosis J32 or J33 OR no EHR “Chronic rhinosinusitis”)
  ‐ RARS (recurrent ARS; ≥ 2 visits with diagnosis J01. AND there was no diagnosis J32 or J33 OR no EHR “Chronic rhinosinusitis”
  ‐ CRS AE (CRS with acute exacerbation; J01 + J32. No J33)
  ‐ CRSwNP AE (J01 + J33)
- Comorbidities (*n* = 1): any doctor‐diagnosed asthma (J45.). This included allergic asthma (J45.0), non‐allergic asthma (J45.1), and non‐specific asthma (J45.9).
- Allergy (*n* = 1): J45.0, or J30., or EHR “allergy” (see Table E1 in the [Additional file 1])
- Immunodeficiency or suspicion of immunodeficiency (*n* = 1): B20, or D80‐84, or EHR “immunodeficiency”

The data extraction was performed by searching the diagnoses in the visit data and diagnostic data (see Table E2 in the [Additional file 1]). In addition, the patient chart texts were searched directly for the diagnostic code or terms referring to the disease in specified words (see Table E2 in the [Additional file 1]).

Allergic rhinitis diagnosis in EHR was based on a positive skin prick test or serum‐specific immunoglobulin *E* (IgE) results, in addition to typical symptoms. non‐allergic rhinitis diagnosis was based on typical symptoms that are not connected to known allergens and/or there is a lack of positive skin prick tests or serum‐specific IgE results of known allergens that could be related to the symptoms during that season. CRS and CRSwNP were diagnosed according to European Position Paper on Rhinosinusitis and Nasal Polyps.^4^ Doctor‐diagnosed asthma means that asthma medication is reimbursed by the Social Insurance Institution of Finland. For this, asthma diagnosis is based on typical history and asthma symptoms, and findings of lung function test (spirometry and peak expiratory flow (PEF)) of at least 15% improvement with bronchodilator test in spirometry (in forced expiratory flow volume in one second (FEV1) or forced vital capacity (FVC)) and/or recurrent 20% diurnal variation in PEF monitoring or recurrent 15% bronchodilator response in PEF monitoring or positive methacholine challenge test (moderate to severe bronchial hyperresponsiveness), or positive lung‐function test confirmed response to inhaled corticosteroid treatment.^12^ Non‐steroidal anti‐inflammatory drug exacerbated respiratory disease diagnosis was based on a positive patient history of wheeze/cough or naso‐ocular symptoms after intake of NSAID or additionally based on a positive reaction (wheeze and/or naso‐ocular reaction) after acetylsalicylic acid (ASA) provocation test at the hospital.^13^

### 2.3 Information extraction from electronic health records

The information extraction method from the medical reports was based on two separate methods. In the first method, we searched International Statistical Classification of Diseases and Related Health Problems (ICD‐10) codes directly from the clinical chart texts.^14^ If any code related to a particular disease was found, then the patient's disease variable was given the value “True”. If a patient had multiple diagnoses for different diseases, then the patient received a True value for each disease. If the patient had the codes J33 and J31, then the patient received True in both groups.

In the second method, we searched for keywords related to the basic diseases (such as “diabetes”, “NERD”).^15^, ^16^ If a keyword was found from the clinical chart text, then the rule‐based validation was conducted. The keywords for text mining are shown in Table E1 of the [Additional file 1]. Figures E1a and E1b of the [Additional file 1] present example steps of the information extraction for the case of diabetes and NERD. In this example, the keywords of diabetes (translated to English) were: ‘diabetes’, ‘sugar’, ‘blood sugar’, and ‘insulin’. The keywords for NERD were: ‘aerd’, ‘samter’, ‘aspirin’, and ‘asa’. When a keyword was found from the clinical text, rule‐based inference identified cases that were related to negation, family history, or good medical status (Column “Rule‐based dictionary” in Table E1 of the [Additional file 1]).

### 2.4 Data analysis

We used Python packages nltk,^17^ scipy,^18^ numpy,^19^ pandas,^20^ and matplotlib‐venn^21^ to implement the data processing, information extraction from clinical text and all statistical analysis. We used *R* packages survival^22^ and glmnet^23^ to model the time to the next visit. Word tokenization for the keyword search was done by the function “tokenization” in the package nltk. Statistical tests were done using the function “stats” in the package scipy. The packages of numpy and pandas were used for data reading and processing. Venn diagrams were performed by using the function “venn3” in the package matplotlib‐venn. We used the function coxph from the package survival for training Cox's proportional hazards models for modeling time to the next visit. The number of previous visits and background variables was used as predictors. The package glmnet was used for training the Least Absolute Shrinkage and Selection Operator (LASSO) model for exploring the best predictors for the hazard of the next visit.^24^, ^25^, ^26^ The parameter *λ* of LASSO was searched for by cross‐validation and 1 standard error from the minimum *λ* value was used.^23^

## 3 RESULTS

### 3.1 The characteristics of patients with rhinitis/rhinosinusitis

Table E3 in the [Additional file 1] presents the characteristics of all patients. The mean age of the patients was 33.6 ± 20.7 years, and 56.1% were female. The follow‐up times did not differ between the groups (data not shown). The mean follow‐up time of adults was 8.6 years and in children (<18 years) it was 8.0 years. The difference was statistically significant (*p* < 0.001). The relative proportion of diagnoses J30., J31., J32., J33, J01., reflecting patients with AR, NAR, CRSsNP, CRSwNP, and ARS, were 33.5%, 27.5%, 20.7%, 10.9%, and 27.2%, respectively (Table E3). Table E4 in the [Additional file 1] presents cross‐tabulation of J30, J31, J32, J33, and J01 patients (number of patients). The patients with J30 (AR) diagnosis were the youngest (20.2 ± 17.5 years) and had the lowest number of visits during the whole follow up time (2.8 ± 6.2) (Table E3). The highest number of visits during the whole follow up time was in the J33 (CRSwNP) diagnosis group (10.2 ± 14.3). The CRSwNP patients were also the oldest (47 ± 15.9 years) and mostly male (60.5%), whereas other diagnosis groups had a predominance of female sex (Table E3).

Table 1 presents the characteristics of the rhinosinusitis subgroups. The relative proportion of CRSsNP, CRSwNP, ARS, RARS, any CRS with acute exacerbation (AE) and, CRSwNP AE were 17.8%, 10.9%, 14.9%, 3.5%, 5.1% and 1.4%, respectively (Table 1). Table E5 in the [Additional file 1] presents cross‐tabulation of any CRS, CRSsNP, CRSwNP, ARS, RARS, any CRS AE and CRSwNP AE patients (number of patients). All groups had female predominance except the CRSwNP subgroup (Table 1). The mean age was the lowest in the ARS group and the highest in the CRSwNP group (Table 1). The mean age was lower among women than in men except in subgroups with CRSwNP or RARS (Table 1).

**TABLE 1:** Characteristics of all patients and with (any) CRS, CRSsNP, CRSwNP, ARS, RARS, CRS AE, CRSwNP AE

Variables	All patients	Any CRS	CRSsNP	CRSwNP	ARS	RARS	Any CRS AE	CRSwNP AE
Number of patients (%)	5080 (100.0)	1603 (31.6)	907 (17.9)	554 (10.9)	759 (14.9)	179 (3.5)	260 (5.1)	72 (1.4)
Female, *n* (%)	2848 (56.1)	942 (58.8)	640 (70.6)	219 (39.5)	492 (64.8)	113 (63.1)	193 (74.2)	41 (56.9)
Age, mean (±SD)	33.6 (20.7)	42.6 (17.1)	39.9 (17.5)	47.0 (15.9)	36.2 (19.2)	38.4 (18.8)	38.3 (17.7)	42.0 (16.8)
Age, men, mean (±SD)	31.0 (17.2)	43.2 (17.2)	37.7 (17.2)	47.6 (17.2)	34.6 (17.2)	39.9 (17.2)	34.2 (17.2)	37.3 (17.2)
Age, women, mean (±SD)	35.6 (17.1)	42.2 (17.1)	40.8 (17.1)	46.1 (17.1)	37.0 (17.1)	37.5 (17.1)	39.8 (17.1)	45.5 (17.1)
Age 0–17, *n* (%)	1350 (26.6)	109 (6.8)	89 (9.8)	15 (2.7)	140 (18.4)	24 (13.4)	30 (11.5)	4 (5.6)
Number of visits*, mean (±SD)	5.1 (8.8)	9.0 (11.9)	7.8 (9.3)	10.2 (14.3)	3.0 (5.7)	7.8 (14.0)	12.6 (14.5)	18.4 (20.4)
Number of visits*, Pulmonology and allergy, mean (±SD)	0.1 (1.0)	0.1 (1.1)	0.1 (1.2)	0.1 (1.1)	0.1 (1.2)	0.4 (3.2)	0.3 (2.2)	0.3 (1.2)
Number of visits*, Pulmonology, mean (±SD)	1.6 (5.3)	2.2 (6.5)	1.9 (6.0)	2.8 (7.2)	1.1 (4.2)	2.3 (10.2)	2.7 (7.5)	4.8 (11.2)
Number of visits*, ENT, mean (±SD)	3.4 (5.8)	6.6 (8.4)	5.8 (6.3)	7.3 (10.5)	1.8 (3.0)	5.1 (5.3)	9.7 (9.9)	13.3 (14.9)
Time interval between visits, days, mean (±SD)	227.1 (321.2)	205.3 (256.6)	212.6 (285.0)	196.6 (217.6)	219.8 (403.3)	168.0 (291.1)	179.4 (253.8)	214.6 (181.0)
Frequency of visits (visits/year, from first to the last visit), mean (±SD)	10.2 (35.6)	7.3 (15.6)	8.7 (18.6)	5.3 (7.6)	23.5 (65.1)	43.4 (63.6)	11.7 (23.9)	3.7 (3.3)
Frequency of visits (visits/year, from first visit to end), mean (±SD)	0.7 (1.2)	1.2 (1.5)	1.1 (1.3)	1.3 (1.6)	0.4 (0.8)	1.0 (2.0)	1.6 (1.7)	2.0 (1.9)
Follow‐up time (days), mean (±SD)	3103 (1257)	3133 (1275)	3125 (1276)	3175 (1267)	3252 (1204)	3235 (1210)	3243 (1272)	3463 (1105)
Diabetes, *n* (%)	535 (10.5)	192 (12.0)	90 (9.9)	79 (14.3)	61 (8.0)	18 (10.1)	30 (11.5)	12 (16.7)
**Chronic respiratory diseases, n (%)**	**1957 (38.5)**	**545 (34.0)**	**280 (30.9)**	**211 (38.1)**	**158 (20.8)**	**39 (21.8)**	**86 (33.1)**	**35 (48.6)**
Obesity, *n* (%)	510 (10.0)	173 (10.8)	96 (10.6)	62 (11.2)	52 (6.9)	14 (7.8)	25 (9.6)	7 (9.7)
Mental disorders, *n* (%)	960 (18.9)	240 (15.0)	147 (16.2)	71 (12.8)	168 (22.1)	40 (22.3)	48 (18.5)	14 (19.4)
Memory disorders, *n* (%)	129 (2.5)	58 (3.6)	30 (3.3)	22 (4.0)	16 (2.1)	5 (2.8)	9 (3.5)	5 (6.9)
Cardiovascular diseases, *n* (%)	1815 (35.7)	650 (40.5)	359 (39.6)	229 (41.3)	253 (33.3)	73 (40.8)	108 (41.5)	34 (47.2)
Cancer, *n* (%)	513 (10.1)	217 (13.5)	107 (11.8)	94 (17.0)	102 (13.4)	31 (17.3)	37 (14.2)	13 (18.1)
Musculoskeletal diseases, *n* (%)	1950 (38.4)	724 (45.2)	438 (48.3)	215 (38.8)	336 (44.3)	89 (49.7)	143 (55.0)	39 (54.2)
Allergy, *n* (%)	2590 (51.0)	582 (36.3)	317 (35.0)	204 (36.8)	119 (15.7)	34 (19.0)	102 (39.2)	43 (59.7)
**Asthma, n (%)**	**2257 (44.4)**	**640 (39.9)**	**301 (33.2)**	**269 (48.6)**	**168 (22.1)**	**42 (23.5)**	**100 (38.5)**	**51 (70.8)**
**NERD, n (%)**	**197 (3.9)**	**142 (8.9)**	**30 (3.3)**	**98 (17.7)**	**18 (2.4)**	**2 (1.1)**	**10 (3.8)**	**16 (22.2)**
Immunodeficiency, *n* (%)	25 (0.5)	16 (1.0)	12 (1.3)	1 (0.2)	4 (0.5)	1 (0.6)	5 (1.9)	0 (0.0)
Iimmunodeficiency or its suspicion, *n* (%)	114 (2.2)	65 (4.1)	44 (4.9)	9 (1.6)	11 (1.4)	10 (5.6)	24 (9.2)	4 (5.6)
Obstr. Sleep apnea, *n* (%)	480 (9.4)	171 (10.7)	77 (8.5)	76 (13.7)	34 (4.5)	11 (6.1)	18 (6.9)	10 (13.9)
Mouth breathing, *n* (%)	345 (6.8)	98 (6.1)	55 (6.1)	33 (6.0)	14 (1.8)	6 (3.4)	12 (4.6)	5 (6.9)
Gastroesophageal reflux, *n* (%)	280 (5.5)	113 (7.0)	71 (7.8)	31 (5.6)	38 (5.0)	9 (5.0)	20 (7.7)	7 (9.7)
COM, *n* (%)	314 (6.2)	60 (3.7)	36 (4.0)	16 (2.9)	38 (5.0)	14 (7.8)	17 (6.5)	3 (4.2)
Tonsils diseases, *n* (%)	323 (6.4)	97 (6.1)	70 (7.7)	18 (3.2)	44 (5.8)	10 (5.6)	25 (9.6)	6 (8.3)
Rhinitis/rhinosinusitis, ≥ 2, *n* (%)	859 (16.9)	658 (41.0)	417 (46.0)	173 (31.2)	87 (11.5)	35 (19.6)	260 (100.0)	72 (100.0)
Other diseases, 0 diseases, *n* (%)	529 (10.4)	183 (11.4)	108 (11.9)	61 (11.0)	184 (24.2)	34 (19.0)	25 (9.6)	1 (1.4)
Other diseases, 1 disease, *n* (%)	**913 (18.0)**	322 (20.1)	202 (22.3)	100 (18.1)	179 (23.6)	35 (19.6)	49 (18.8)	8 (11.1)
Other diseases, 2 diseases, *n* (%)	**894 (17.6)**	316 (19.7)	182 (20.1)	113 (20.4)	145 (19.1)	29 (16.2)	44 (16.9)	14 (19.4)
Other diseases, 3 diseases, *n* (%)	**864 (17.0)**	198 (12.4)	108 (11.9)	65 (11.7)	73 (9.6)	36 (20.1)	37 (14.2)	7 (9.7)
Other diseases, ≥ 4 diseases, *n* (%)	**1880 (37.0)**	584 (36.4)	307 (33.8)	215 (38.8)	178 (23.5)	45 (25.1)	105 (40.4)	42 (58.3)
Number of any diseases, 1 disease, *n* (%)	486 (9.6)	142 (8.9)	74 (8.2)	56 (10.1)	177 (23.3)	31 (17.3)	0 (0.0)	0 (0.0)
Number of any diseases, 2 diseases, *n* (%)	824 (16.2)	253 (15.8)	156 (17.2)	86 (15.5)	168 (22.1)	33 (18.4)	23 (8.8)	1 (1.4)
Number of any diseases, 3 diseases, *n* (%)	876 (17.2)	302 (18.8)	178 (19.6)	98 (17.7)	143 (18.8)	29 (16.2)	43 (16.5)	7 (9.7)
Number of any diseases, 4 diseases, *n* (%)	889 (17.5)	233 (14.5)	133 (14.7)	81 (14.6)	86 (11.3)	30 (16.8)	45 (17.3)	14 (19.4)
Number of any diseases, ≥ 5 diseases, *n* (%)	2005 (39.5)	673 (42.0)	366 (40.4)	233 (42.1)	185 (24.4)	56 (31.3)	149 (57.3)	50 (69.4)
*During the whole follow up time								

*Note*: Some of the most prevalent co‐morbidities are marked in bold text.

Abbreviations: ARS, Acute purulent rhinosinusitis; COM, Chronic otitis media; CRS AE, CRS Acute exacerbation; CRS, Chronic rhinosinusitis; CRSsNP, CRS without nasal polyps; CRSwNP, CRS with nasal polyps; ENT, Ear nose throat diseases; NAR, Nonallergic rhinitis; NERD, Patient‐reported non‐steroidal anti‐inflammatory drug ‐exacerbated respiratory disease; Other diseases ≠ AR, NAR, ARS, CRS; RARS, Recurrent ARS; Rhinitis/rhinosinusitis = AR, NAR, ARS, or CRS; SD, Standard deviation.

**FIGURE 1:**
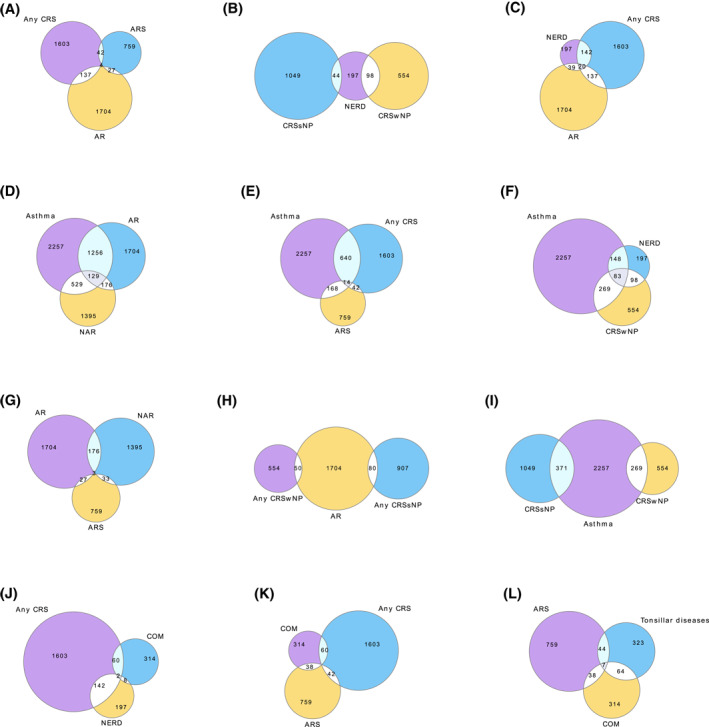
Venn diagrams show the absolute number of subjects having overlapping diagnoses of rhinitis/rhinosinusitis and closely related diseases. Note that NERD is not an independent disease but is associated with asthma and/or CRS. AR, Allergic rhinitis; ARS, Acute purulent rhinosinusitis; COM, Chronic otitis media; CRS, Chronic rhinosinusitis; CRSsNP, CRS without nasal polyps; CRSwNP, CRS with nasal polyps; NAR, Nonallergic rhinitis; NERD, non‐steroidal anti‐inflammatory drug ‐exacerbated respiratory disease

We showed a high overlap of upper airway diagnoses of rhinitis/rhinosinusitis patients (Table E3, Table 1, Figure 1 Venn‐diagrams). At least one other comorbidity/ies than rhinitis/rhinosinusitis was detected in 89.6% of cases (Table 1, E3). Overall, the most common comorbidities were asthma (44.4%), other chronic respiratory diseases (38.5%), musculoskeletal diseases (38.4%), and cardiovascular diseases (35.7%) (Table 1).

The relative proportion of comorbid asthma and allergy were 44.4% and 51.0%, respectively (Table E3). The relative proportion of comorbid asthma among patients with AR, NAR, CRSsNP, CRSwNP, and ARS was 73.7%, 37.9%, 35.4%, 48.6%, and 27.6%, respectively (Table E3). The relative proportion of allergy as comorbidity among patients with AR, NAR, CRSsNP, CRSwNP, and ARS was 100%, 46.0%, 36.0%, 36.8%, and 22.5%, respectively (Table E3). The relative proportion of comorbid NERD was 3.9% in all patients, and among patients with AR, NAR, CRSsNP, CRSwNP, and ARS it was 2.3%, 3.4%, 4.2%, 17.7%, and 3.5%, respectively (Table E3). Venn diagrams show the absolute number of subjects having overlapping diagnoses of rhinitis/rhinosinusitis and closely related diseases (Figure 1).

When observing the rhinosinusitis subgroups in Table 1, the relative proportion of asthma in any CRS, CRSsNP, CRSwNP, ARS, RARS, any CRS AE and CRSwNP AE groups were 39.9%, 33.2%, 48.6%, 22.1%, 23.5%, 38.5% and 70.8%, respectively. The relative proportion of allergy in any CRS, CRSsNP, CRSwNP, ARS, RARS, any CRS AE and CRSwNP AE groups were 36.3%, 35.0%, 36.8%, 15.7%, 19.0%, 39.2% and 59.7%, respectively (Table 1). The relative proportion of NERD in any CRS, CRSsNP, CRSwNP, ARS, RARS, any CRS AE and CRSwNP AE groups were 8.9%, 3.3%, 17.7%, 2.4%, 1.1%, 3.9% and 22.2%, respectively (Table 1).

Comorbid chronic respiratory diseases (other than asthma) were more frequent among NAR and AR patients than among CRS patients (Table E3). CRSwNP group had the highest relative proportion of cardiovascular diseases (41.3%), diabetes (14.3%), and obstructive sleep apnea (13.7%) (Table E3). Musculoskeletal diseases existed in 38.4%, mental disease(s) in 18.9, cancer in 10.1%, obesity in 10.0%, Chronic otitis media (COM) in 6.2% and tonsillar disease(s) in 6.4% of cases. The relative proportion of diabetes was second highest in the NAR group (12.3%) and it was similar in the other groups, AR, CRSsNP, and ARS (Table E3).

When observing the rhinosinusitis groups in Table 1, the most frequent other comorbidities were other chronic respiratory diseases (48.6%), musculoskeletal diseases (46.7%), and cardiovascular diseases (40.5%). CRSwNP AE subgroup showed the highest relative proportion of asthma (70.8%), allergy (59.7%), musculoskeletal diseases (54.2%), cardiovascular diseases (47.2%), NERD (22.2%), cancer (18.1%), diabetes (16.7%), gastroesophageal reflux disease (9.7%), immunodeficiency or its suspicion (9.2%), mouth breathing (6.94%), memory diseases (6.9%), in comparison to other subgroups (Table 1).

### 3.2 The frequency of outpatient visits for rhinitis/rhinosinusitis

The mean (±standard deviation) follow‐up time for the patients in our study was 8.5 ± 3.4 years (Table E3). The mean count of the visits during the whole follow up time was 5.1 ± 8.8 visits and the average time between two visits was 227 ± 321 days. The patients with the diagnosis J33 had the highest number of visits during the whole follow up time (10.2 ± 14.3 visits). Furthermore, their time interval between two visits was the shortest (196.6 ± 217.6 days). All groups attended most of their visits to the Ear nose throat diseases (ENT) department, although in the J30 (AR) group the difference in the number of visits between the ENT and pulmonology departments was small (1.5 vs. 1.3) (data not shown). The time interval between visits was calculated in cases with >1 visit. This was similar in all groups (data not shown). A higher number of visits correlated with shorter intervals (data not shown).

**FIGURE 2:**
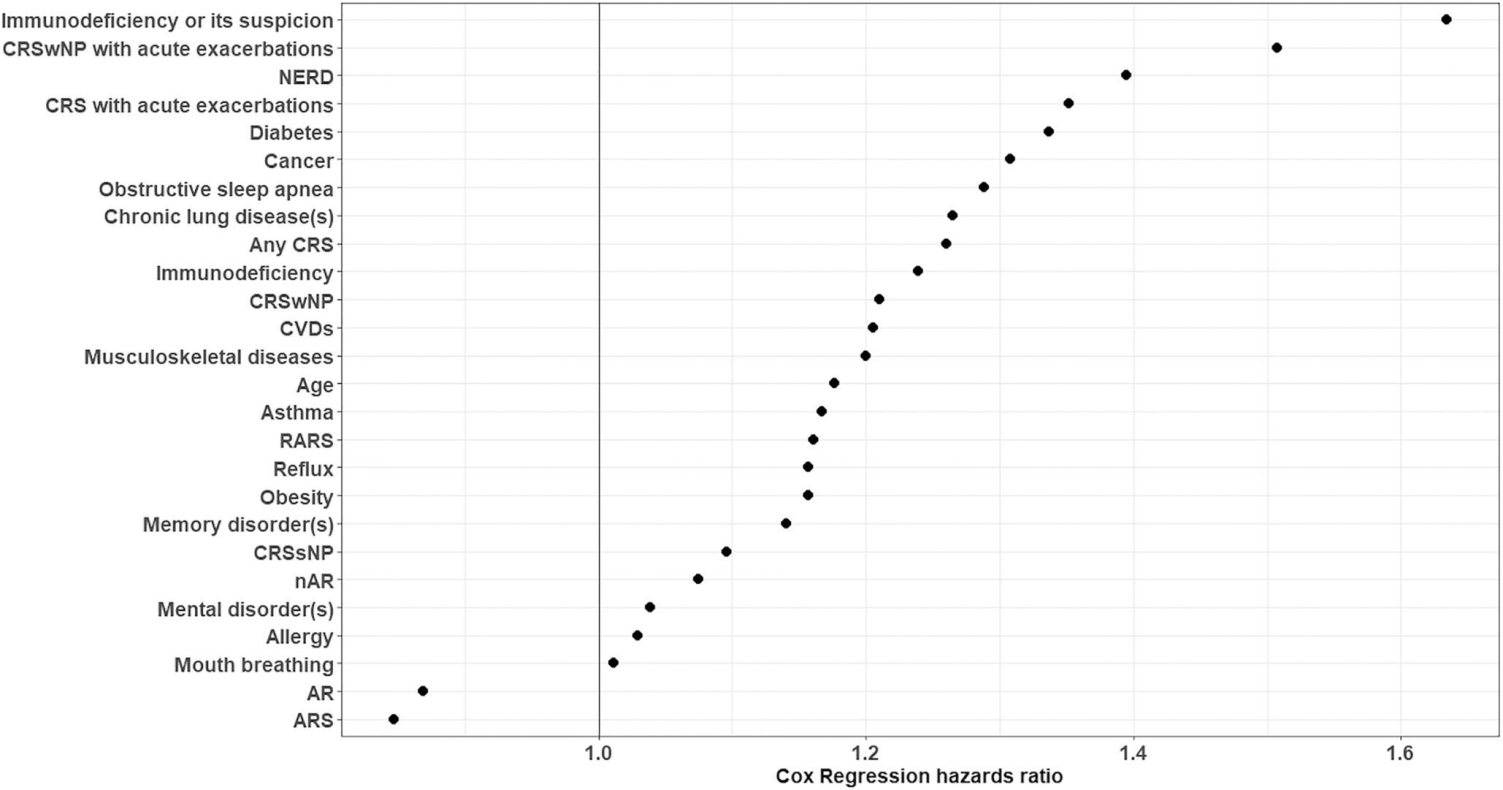
Forest plot of Cox regression hazards ratios. Cox's proportional hazards model modeled time to next visit. The number of previous visits and background variables were used as predictors. AR, Allergic rhinitis; ARS, Acute purulent rhinosinusitis; CRS, Chronic rhinosinusitis; CRSsNP, CRS without nasal polyps; CRSwNP, CRS with nasal polyps; CVDs, Cardiovascular diseases; nAR, Nonallergic rhinitis; NERD, Patient‐reported non‐steroidal anti‐inflammatory drug ‐exacerbated respiratory disease; RARS, Recurrent ARS

We modeled the time to the next visit with Cox's proportional hazards model (Figure 2). We used the number of previous visits and background variables as predictors. In models, the following 23 variables were positively associated with visit frequency: age, diabetes, chronic lung disease(s), obesity, mental disorder(s), memory disorder(s), CVDs, cancer, musculoskeletal diseases, allergy, asthma, NERD, immunodeficiency, immunodeficiency or its suspicion, obstructive sleep apnea, NAR, any CRS, CRSsNP, CRSwNP, RARS, CRS with acute exacerbations, CRSwNP with acute exacerbations and reflux (Figure 2). The following variables were negatively associated with visit frequency, AR, and ARS. Of all variables, only gender and mouth breathing were not associated with visit frequency (Figure 2). Figure E2 of the [Additional file 1] illustrates time survival to the next visit for different variables.

With the LASSO model, we found that the visit frequency risk increased with the number of upper airway diseases; as compared to 1 disease, adjusted HR (coef) was 1.099 (0.09).

## 4 DISCUSSION

We found a strong overlap of upper respiratory diseases. The most common comorbidities were other chronic respiratory diseases but also musculoskeletal and cardiovascular diseases. Comorbidities were associated with a high outpatient visit burden.

We detected that AR/NAR/ARS/CRS diagnoses were co‐existing in about fifth of the present cases. Previous studies have confirmed the overlapping of these conditions,^2^, ^3^, ^4^ although they differ in etiopathology, risk factors, and clinical picture.

We showed here that more than a third of rhinitis/rhinosinusitis patients have asthma, and other chronic respiratory, musculoskeletal and/or cardiovascular diseases as comorbidities. The most frequent comorbidity was asthma (about 40%). This is in line with previous studies showing that AR, CRS, and NERD are common comorbidities of asthma and vice versa.^4^ Previous studies of our group and other groups have shown that asthma and NERD are common comorbidities of CRS and that the upper and lower airway diseases exacerbate each other.^4^, ^27^ Recalcitrant CRS increases the incidence of asthma. It seems that patients with comorbid CRS and asthma ± NERD would benefit from a customized treatment plan and follow‐up beyond the first surgery to achieve better long‐term outcomes.^27^ Non‐steroidal anti‐inflammatory drug exacerbated respiratory disease is associated with unknown pathobiology and increased morbidity.^7^, ^8^, ^9^ There is little previous knowledge of the prevalence of NERD. In the present study, the relative proportion of NERD was 3.88% in all patients and 17.7% in the CRSwNP group. This is in line with previous studies showing that the prevalence of NERD among CRSwNP patients is 16%.^10^ A systematic review showed that the prevalence of NERD was about 10% among patients with CRSwNP and 17% among asthmatics.^28^ Increased awareness of the condition will likely improve prevalence estimates of NERD.

We showed a high relative proportion of obstructive sleep apnea, especially among NAR and CRSwNP groups. Literature has shown that in patients with obstructive sleep apnea, nasal symptoms (namely obstruction) and thus ENT consultations are frequent,^29^, ^30^ which may in part explain the high proportion.

Overall, we demonstrated a high relative proportion of other chronic respiratory diseases (about 40%) in this patient population. In line with this, literature shows that up to two‐thirds of patients with CRS are affected by comorbid asthma,^4^ chronic obstructive pulmonary disease,^31^ or bronchiectasis.^32^, ^33^ CRS patients have decreased lung function regardless of the presence of asthma.^34^ Rhinitis/rhinosinusitis is the most prevalent comorbidity of asthma, and CRS has shown to increase asthma risk and vice versa.^4^, ^35^

Other common comorbidities of our study population were musculoskeletal diseases (38.4%), cardiovascular diseases (35.7%), and mental disease(s) (18.9%). A 10th had co‐existing cancer, and/or obesity. The population‐based global age‐standardized rate of musculoskeletal disorders has been reported to be 210 per 1000, and 24 for mental disorders, 15 for chronic respiratory diseases, 4.6 for CVDs, and 2.2 for neoplasms.^36^ It could be speculated that the high relative proportion of these general diseases in our hospital cohort might be only in part due to the high burden of these diseases in the general population,^36^, ^37^ and could in part be related to shared etiology. There is limited knowledge of the association of common non‐communicable diseases with inflammatory upper airway diseases and thus further studies are still needed.

Interestingly, CRSwNP with the acute exacerbations (AE) subgroup showed the highest relative proportion in most of the other diseases. There is only little literature on the syndromic nature of CRSwNP (except asthma or NERD) and therefore further epidemiological evaluation is warranted to reduce suffering and costs.^38^

Immunodeficiency or its suspicion existed in 10th of the cases in the CRS AE group, and 2.2% of all subjects, reflecting that especially the CRS AE group, for example, patients suffering from recurrent acute (infectious) exacerbations of CRS, are under suspicion of immunodeficiency. True immunodeficiency diagnoses existed only in 0.5% of cases, and 1.9% of the cases within the CRS AE group. Immunodeficiency and granulomatosis with polyangiitis have been shown to increase the revision endoscopic sinus surgery risk.^39^ Although the variable “suspicion of immunodeficiency” is different from diagnosed immunodeficiency, it might indirectly reflect the comparable situation with poor CRS control, which makes the physician suspect this rare comorbidity.

We showed that all diseases except three (AR, acute rhinosinusitis, and mouth breathing), were associated with a high visit burden. The number of inflammatory upper airway diseases increased the risk of visit burden. This result could help in patient counseling and planning of treatment processes. We have previously shown that patients with at least one chronic disease have an increased risk of severe asthma.^9^ Multiple chronic conditions have been shown to increase the risk of physician visit frequency.^11^ Also, AR burden in primary care has been shown to increase visit burden^40^ as well as pediatric acute rhinosinusitis in hospital care.^41^ Comorbid CRS has been shown to increase asthma‐related emergency visits.^42^

The data showed that extracting EHR data from the selected variables worked well in this type of study. The accuracy of EHR data extraction has previously been shown for example, in joint implant registries.^43^ The limitation of EHR extraction method is that physicians may make EHR entries in different ways, or some information may not be found in the EHR at all. This source of bias was minimized by extracting data of a random sample of patients over a long period of time, from different physicians, and from a long follow‐up period.

The strengths of this study include a large and random sample of patients with outpatient visits and the use of text mining of EHR texts, in addition to coded diagnoses. We showed that information extraction of EHR shows high performance in finding NERD patients as well as non‐respiratory comorbidities of patients with rhinologic diseases. The retrospective character, selected hospital population and potential inadequate data extraction due to insufficient coding put some limitations on the study. The information about elsewhere visits such as general practitioners, occupational healthcare, or the private sector was not available. We acknowledge that the control group, data of symptom scores, medications, polyp scores, Lund‐Mackay scores, etc. were not available in this study. The role of sinus surgery has been analyzed elsewhere.^44^ Relative proportion is not fully corresponding to prevalence, which may explain different results compared to general population studies. Some diagnoses, such as first J31 and later J30, may have been used in the same patient before and after allergy test results. The physician sometimes enters only one diagnosis (J30) in cases of mixed rhinitis (J30 & J31), so in real life, the proportion of co‐existing J30 and J31 diagnoses is likely to be higher. Local allergic rhinitis (LAR) is also caused by IgE‐mediated reaction, but due to the lack of validated diagnostic tests, NAR diagnoses might also include LAR cases. Hence the findings need validation in other populations. In Finland, there are excellent EHR also in the basic healthcare, private sector, and occupational health, and similar analysis in these populations would provide valuable information about the overall disease burden.

## 5 CONCLUSIONS

A strong overlap of diseases occurs in the care of AR, NAR, ARS, CRSwNP, and CRSsNP. The most common comorbidities are asthma, other chronic respiratory diseases, musculoskeletal diseases, and cardiovascular diseases. CRSwNP with acute exacerbations group has the highest relative proportion of comorbidities. Comorbidities and the number of inflammatory upper airway diseases are associated with the outpatient visit burden for chronic rhinitis or rhinosinusitis. Non‐steroidal anti‐inflammatory drug exacerbated respiratory disease exists in 4% of all patients and 18% of the CRSwNP group. Active management of syndromic upper airway diseases could have an advantageous socio‐economic impact.

## AUTHOR CONTRIBUTIONS

**Mikko Nuutinen**: Conceptualization; Equal, Data curation; Lead, Formal analysis; Lead, Investigation; Equal, Methodology; Lead, Visualization; Lead, Writing–original draft; Equal, Writing–review & editing; Equal, **Annina Lyly**: Conceptualization; Equal, Investigation; Equal, Writing–original draft; Equal, Writing–review & editing; Equal, **Paula Virkkula**: Conceptualization; Supporting, Investigation; Supporting, Writing–review & editing; Equal, **Maija Hytonen**: Conceptualization; Supporting, Investigation; Supporting, Writing–review & editing; Equal, **Elmo Saarentaus**: Methodology; Supporting, Validation; Supporting, Writing–review & editing; Supporting, **Antti Makitie**: Conceptualization; Supporting, Project administration; Supporting, Supervision; Supporting, Writing–review & editing; Equal, **Aarno Palotie**: Methodology; Supporting, Supervision; Supporting, Writing–review & editing; Supporting, **Paulus Torkki**: Data curation; Equal, Formal analysis; Equal, Methodology; Equal, Writing–review & editing; Equal, **Jari Haukka**: Conceptualization; Equal, Data curation; Equal, Formal analysis; Equal, Investigation; Equal, Methodology; Equal, Validation; Equal, Visualization; Equal, Writing–review & editing; Equal, **Sanna Toppila‐Salmi**: Conceptualization; Lead, Data curation; Supporting, Formal analysis; Equal, Funding acquisition; Lead, Investigation; Equal, Methodology; Equal, Project administration; Lead, Resources; Lead, Supervision; Lead, Validation; Equal, Visualization; Supporting, Writing–original draft; Equal, Writing–review & editing; Equal.

## CONFLICT OF INTERESTS

Sanna Toppila‐Salmi report consultancies for ALK‐Abelló, AstraZeneca, ERT, GlaxoSmithKline, Novartis, Sanofi, Roche Products, and a grant from GlaxoSmithKline. Annina Lyly reports consultancies for Viatris, Novartis and Sanofi. Paula Virkkula reports consultancies for Sanofi and GlaxoSmithKline. All are outside the submitted work. All other authors declare no conflicts of interest.

## CONSENT FOR PUBLICATION

Not applicable.

## Supporting information

Supplementary Material

## Data Availability

The data that support the findings of this study are available from Helsinki University Hospital but restrictions apply to the availability of these data, which were used under license for the current study, and so are not publicly available. Data are however available from the authors upon reasonable request and with permission of Helsinki University Hospital.

## References

[1] Hastan DF , Fokkens WJ , Bachert C , et al. Chronic rhinosinusitis in Europe–an underestimated disease. A GA2LEN study. Allergy. 2011;66(9):1216‐1223. 10.1111/j.1398-9995.2011.02646.x 21605125

[2] Bousquet J , Anto JM , Bachert C , et al. Allergic rhinitis. Nat Rev Dis Prim. 2020;6(1):1‐7. 10.1038/s41572-020-00227-0 33273461

[3] Hellings PW , Klimek L , Cingi CE , et al. Non‐allergic rhinitis: position paper of the European academy of allergy and clinical Immunology. Allergy. 2017;72(11):1657‐1665. 10.1111/all.13200 28474799

[4] Fokkens WJ , Lund VJ , Hopkins C , et al. European position paper on rhinosinusitis and nasal polyps 2020. Rhinology. 2020;58(Suppl S29):1‐464. Published 2020 Feb 20. 10.4193/Rhin20.600 32077450

[5] Lourijsen ES , Fokkens WJ , Reitsma S . Direct and indirect costs of adult patients with chronic rhinosinusitis with nasal polyps. Rhinology. 2020;16(0):0. 10.4193/rhin19.468 32415826

[6] Bousquet J , Fokkens W , Burney P , et al. Important research questions in allergy and related diseases: nonallergic rhinitis: a GA2LEN paper. Allergy. 2008;63(7):842‐853. 10.1111/j.1398-9995.2008.01715.x 18588549

[7] Kowalski ML , Agache I , Bavbek S , et al. Diagnosis and management of NSAID‐exacerbated respiratory disease (N‐ERD)—a EAACI position paper. Allergy. 2019;74(1):28‐39. 10.1111/all.13599 30216468

[8] Lyly A , Laulajainen‐Hongisto A , Turpeinen H , et al. Factors affecting upper airway control of NSAID‐exacerbated respiratory disease: a real‐world study of 167 patients. Immun Inflamm Dis. 2021;9(1):80‐89. 10.1002/iid3.347 33400396 PMC7860608

[9] Toppila‐Salmi S , Lemmetyinen R , Chanoine S , et al. Risk factors for severe adult‐onset asthma: a multi‐factor approach. BMC Pulm Med. 2021;21(1):214. 10.1186/s12890-021-01578-4 34238263 PMC8268541

[10] Stevens WW , Peters AT , Hirsch AG , et al. Clinical characteristics of patients with chronic rhinosinusitis with nasal polyps, asthma, and aspirin‐exacerbated respiratory disease. J Allergy Clin Immunol Pract. 2017;5(4):1061‐1070. e3. 10.1016/j.jaip.2016.12.027 28286156 PMC5503772

[11] Hessel A , Gunzelmann T , Geyer M , Brähler E . Inanspruchnahme medizinischer Leistungen und Medikamenteneinnahme bei über 60jährigen in Deutschland–gesundheitliche, sozialstrukturelle, sozio‐demographische und subjektive Faktoren. Z Gerontol Geriatr. 2000;33(4):289‐299. 10.1007/s003910070049 11028281

[12] Global Initiative for Asthma . Global Strategy for Asthma Management and Prevention; 2020. www.ginasthma.org

[13] Li KL , Lee AY , Abuzeid WM . Aspirin exacerbated respiratory disease: epidemiology, pathophysiology, and management. Med Sci. 2019;7(3):45. 10.3390/medsci7030045 PMC6473909 30884882

[14] World Health Organization . International Statistical Classification of Diseases and Related Health Problems (ICD); 2021. https://www.who.int/standards/classifications/classification‐of‐diseases

[15] Baus A , Hendryx M , Pollard C . Identifying patients with hypertension: a case for auditing electronic health record data. Perspect Health Inf Manag. 2012;9(Spring):1e. https://www.ncbi.nlm.nih.gov/pmc/articles/PMC3329209/ PMC3329209 22737097

[16] Friedlin J , Grannis S , Overhage JM . Using natural language processing to improve accuracy of automated notifiable disease reporting. AMIA Annu Symp Proc. 2008;2008:207‐211. https://www.ncbi.nlm.nih.gov/pmc/articles/PMC2656046/ PMC2656046 18999177

[17] Bird S , Klein E , Loper E . Natural Language Processing with Python. 1st ed. O’Reilly Media, Inc.; 2009.

[18] Virtanen P , Gommers R , Oliphant TE , et al. SciPy 1.0: fundamental algorithms for scientific computing in Python. Nat Methods. 2020;17(3):261‐272. 10.1038/s41592-019-0686-2 32015543 PMC7056644

[19] van der Walt S , Colbert SC , Varoquaux G . The NumPy array: a structure for efficient numerical computation. Comput Sci Eng. 2011;13(2):22‐30. 10.1109/mcse.2011.37

[20] McKinney W . Data structures for statistical computing in Python. In: van der Walt S Millman J , eds. Proceedings of the 9th Python in Science Conference; 2010:56‐61.

[21] Tretyakov K . Matplotlib‐Venn – functions for plotting area‐proportional two‐ and three‐way Venn diagrams in matplotlib. Python Package Version 0.11.6. 2020. https://pypi.org/project/matplotlib‐venn/

[22] Therneau TM , Grambsch PM . Modeling Survival Data: Extending the Cox Model. 0‐387‐98784‐3. Springer; 2000.

[23] Friedman J , Hastie T , Tibshirani R . Regularization paths for generalized linear models via coordinate descent. J Stat Software. 2010;33(1):1. 10.18637/jss.v033.i01 PMC2929880 20808728

[24] Simon N , Friedman J , Hastie T , Tibshirani R . Regularization paths for Cox’s proportional hazards model via coordinate descent. J Stat Software. 2011;39(5):1. 10.18637/jss.v039.i05 PMC4824408 27065756

[25] Simon N , Friedman J , Hastie T . A blockwise descent algorithm for group‐penalized multiresponse and multinomial regression. arXiv preprint arXiv:1311.6529. 2013.

[26] Tibshirani R , Bien J , Friedman J , et al. Strong rules for discarding predictors in lasso‐type problems. J Roy Stat Soc B. 2012;74(2):245‐266. 10.1111/j.1467-9868.2011.01004.x PMC4262615 25506256

[27] Penttilä E , Sillanpää S , Vento SI , et al. Eosinophilia, asthma, NERD and the use of oral corticosteroids predict uncontrolled chronic rhinosinusitis with nasal polyps after surgery. Asian Pac J Allergy Immunol. 2021. Epub ahead of print. PMID: 34542306. 10.12932/AP-310321-1102 34542306

[28] Rajan JP , Wineinger NE , Stevenson DD , White AA . Prevalence of aspirin‐exacerbated respiratory disease among asthmatic patients: a meta‐analysis of the literature. J Allergy Clin Immunol. 2015;135(3):676‐681. 10.1016/j.jaci.2014.08.020 25282015

[29] Young T , Evans L , Finn L , Palta M . Estimation of the clinically diagnosed proportion of sleep apnea syndrome in middle‐aged men and women. Sleep. 1997;20(9):705‐706. 10.1093/sleep/20.9.705. https://pubmed.ncbi.nlm.nih.gov/9406321/ 9406321

[30] Kreivi H.‐R , Virkkula P , Lehto JT , Brander PE . Upper airway symptoms in primary snoring and in sleep apnea. Acta Otolaryngol. 2012;132(5):510‐518. 10.3109/00016489.2011.644803. https://pubmed.ncbi.nlm.nih.gov/22217396/ 22217396

[31] Arndal E , Sørensen AL , Lapperre TS , et al. Chronic rhinosinusitis in COPD: a prevalent but unrecognized comorbidity impacting health related quality of life. Respir Med. 2020;171:106092. 10.1016/j.rmed.2020.106092 32846336

[32] Sheng H , Yao X , Wang X , Wang Y , Liu X , Zhang L . Prevalence and clinical implications of bronchiectasis in patients with overlapping asthma and chronic rhinosinusitis: a single‐center prospective study. BMC Pulm Med. 2021;21(1):1‐3. 10.1186/s12890-021-01575-7 34225679 PMC8258939

[33] Peters AT , Bose S , Guo A , et al. Prevalence of bronchiectasis in patients with chronic rhinosinusitis in a tertiary care center. J Allergy Clin Immunol Pract. 2021;24(8):3188‐3195.e2. 10.1016/j.jaip.2021.04.054 PMC11216716 33965595

[34] Kariya S , Okano M , Higaki T , et al. Chronic rhinosinusitis patients have decreased lung function. Inter Forum Allergy Rhinol. 2014;4(10):828‐833. 10.1002/alr.21370 25132678

[35] Ryu G , Min C , Park B , Choi HG , Mo JH . Bidirectional association between asthma and chronic rhinosinusitis: two longitudinal follow‐up studies using a national sample cohort. Sci Rep. 2020;10(1):1‐0. 10.1038/s41598-020-66479-8 32533009 PMC7293248

[36] Cieza A , eCausey K , Kamenov K , Wulf Hanson S , Chatterji S , Vos T . Global estimates of the need for rehabilitation based on the global burden of disease study 2019: a systematic analysis for the global burden of disease study 2019. Lancet. 2020;396(10267):2006‐2017. 10.1016/s0140-6736(20)32340-0. https://pubmed.ncbi.nlm.nih.gov/33275908/ 33275908 PMC7811204

[37] Huijts T , Stornes P , Eikemo TA , Bambra C , Consortium HN . Prevalence of physical and mental non‐communicable diseases in Europe: findings from the European Social Survey (2014) special module on the social determinants of health. Eur J Public Health. 2017;27(Suppl_1):8‐13. 10.1093/eurpub/ckw232 28355647

[38] Bachert C , Bhattacharyya N , Desrosiers M , Khan AH . Burden of disease in chronic rhinosinusitis with nasal polyps. J Asthma Allergy. 2021;14:127‐134. 10.2147/jaa.s290424 33603409 PMC7886239

[39] Miglani A , Divekar RD , Azar A , Rank MA , Lal D . Revision endoscopic sinus surgery rates by chronic rhinosinusitis subtype. Inter Forum Allergy Rhinol. 2018;8(9):1047‐1051. 10.1002/alr.22146 29851243

[40] Price D , Scadding G , Ryan D , et al. The hidden burden of adult allergic rhinitis: UK healthcare resource utilisation survey. Clin Transl Allergy. 2015;5(1):1‐2. 10.1186/s13601-015-0083-6 26583068 PMC4650835

[41] Gilani S , Shin JJ . The burden and visit prevalence of pediatric chronic rhinosinusitis. Otolaryngol Head Neck Surg. 2017;157(6):1048‐1052. 10.1177/0194599817721177 28741448

[42] Gleadhill C , Speth MM , Gengler I , et al. Chronic rhinosinusitis disease burden is associated with asthma‐related emergency department usage. Eur Arch Oto‐Rhino‐Laryngol. 2021;278(1):93‐99. 10.1007/s00405-020-06259-2 32749608

[43] Giori NJ , Radin J , Callahan A , et al. Assessment of Extractability and accuracy of electronic health record data for Joint implant registries. JAMA Netw Open. 2021;4(3):e211728. 10.1001/jamanetworkopen.2021.1728 33720372 PMC7961313

[44] Nuutinen M , Haukka J , Virkkula P , Torkki P , Toppila‐Salmi S . Using machine learning for the personalised prediction of revision endoscopic sinus surgery. PLoS ONE. 2022;17(4):e0267146. PMID: 35486626; PMCID: PMC9053825. 10.1371/journal.pone.0267146 35486626 PMC9053825

