# Supplementary material for: The relative proportion of comorbidities among rhinitis and rhinosinusitis patients and their impact on visit burden
Source: Clin Transl Allergy. 2022 Jul 21;12(7):e12181. doi: 10.1002/clt2.12181 (PMC9301683; doi:10.1002/clt2.12181)
Supplement: Supplementary file 1 — Supplementary Material [file CLT2-12-e12181-s001.docx]

Online Repository: The relative proportion of comorbidities among rhinitis and rhinosinusitis patients and their impact on visit burden

Mikko Nuutinen^1^, Annina Lyly^2,3^, Paula Virkkula^3^, Maija Hytönen^3^, Elmo Saarentaus^3^, Antti Mäkitie^3^, Aarno Palotie^4,5,6,7^, Paulus Torkki^8^, Jari Haukka^8,*^, and Sanna Toppila-Salmi^1,2,9,*^

*Shared last author

^1^Haartman Institute, University of Helsinki, Helsinki, Finland

^2^Skin and Allergy Hospital, University of Helsinki and Helsinki University Hospital, Helsinki, Finland

^3^Department of Otorhinolaryngology - Head and Neck Surgery, Helsinki University Hospital and University of Helsinki, Helsinki, Finland

^4^Institute for Molecular Medicine Finland (FIMM), HiLIFE, University of Helsinki, Helsinki, Finland

^5^Analytic and Translational Genetics Unit, Massachusetts General Hospital, Boston, Massachusetts, USA

^6^Program in Medical and Population Genetics, Broad Institute of Harvard and MIT, Cambridge, Massachusetts, USA

^7^Stanley Center for Psychiatric Research, Broad Institute of Harvard and MIT, Cambridge, Massachusetts, USA

^8^Department of Public Health, University of Helsinki, Finland

^9^Department of Pulmonary Medicine, Heart and Lung Center, Helsinki University Hospital and University of Helsinki, Helsinki, Finland

June 29, 2022

**Table E1:** Key words for the mining of patient variables from the clinical text and disease

specific dictionaries for evaluating when matched key words relate to the negation of disease,

family history or good medical status

| **Disease** | **Key word** | **Rule based dictionary** | **Extra rules** |
| --- | --- | --- | --- |
| Allergy | cat | no, negative, mother, sister |  |
|  | birch | no, negative, normal |  |
|  | prick | no, negative, normal |  |
|  | skin prick test | no, negative, normal |  |
|  | rast | no, negative | Only "rast" or "rast-xxx" \\ |
| Cancer | malignant | no |  |
|  | carcinoma | no |  |
|  | basalioma | no |  |
|  | cancer | mother, father, family |  |
|  | tumor | no, benign |  |
|  | melanoma |  |  |
| Cardiovascular disease | hypertension | no, normal, mother, father, family | negation if in same sentence |
|  | Coronary artery disease | no, normal, mother, father, family | negation if in same sentence |
|  | coronary heart disease | no, normal, mother, father, family | negation if in same sentence |
|  | aneurysm | no, normal, mother, father, family | negation if in same sentence |
|  | cerebral hemorrhage | no, normal, mother, father, family | negation if in same sentence |
|  | heart attack | no, normal, mother, father, family | negation if in same sentence |
|  | angina | no |  |
|  | heart | normal, ok, ordinal, regular, good, balance, |  |
|  |  | family, mother, father, no, clean, |  |
|  |  | impeccable, clean, normal, |  |
|  |  | health, calm, vibrant, symmetric, parents |  |
|  |  | ordinal, okay, normal, compensation |  |
|  | rhythm | regular, steady, calm, silent, no, family |  |
| Chronic respiratory disease | bronchitis | no, mother, father, family |  |
|  | copd | no, mother, father, family |  |
| Diabetes | diabetes | family, father, mother |  |
|  | insuline |  |  |
|  | sugar | normal, ok, father, mother, father, candied, |  |
|  |  | sugar free, food, fat, diet |  |
|  | blood sugar | good, normal, check |  |
| Memory disorder | memory problem | no |  |
|  | memory impairment | no |  |
|  | memory disorder | caregiver, wife, husband, mother |  |
|  | Alzheimer | caregiver, wife, husband, mother |  |
|  | dementia | work, working |  |
| Mental disorder | depression |  |  |
|  | mood disorder |  |  |
|  | adhd |  |  |
|  | psychiatrist | works, job, working |  |
|  | behavioral |  | Same sentence should include: "disorder", \\ |
|  |  |  | "problem", "difficulties", "regulation", |
|  |  |  | agressive", "control" or "self destructive" |
| Musculoskeletal diseases | osteoporosis | no, mother, father, family |  |
|  | joint | no, normal, family, mother, father, |  |
|  |  | calm, good, working, over-moving |  |
|  | tules |  | Only "tules" or "tules-xxx" |
|  | back | no, mother, father, family, close relative | Should be: back pain, back ache, |
|  |  |  | back problem, spinal cord injury, |
|  |  |  | ankylosing spondylitis |
| Obesity | obesity | no, mother, father, family |  |
|  | fat | no, mother, father, family |  |
| NERD | aerd | no |  |
|  | samter | no |  |
|  | aspirin | no |  |
|  | asa | no, mg |  |
| Immunodeficiency or its suspicion | infectious disease doctor | no |  |
|  | susceptibility to infection | no |  |
|  | immune deficiency | no |  |

| 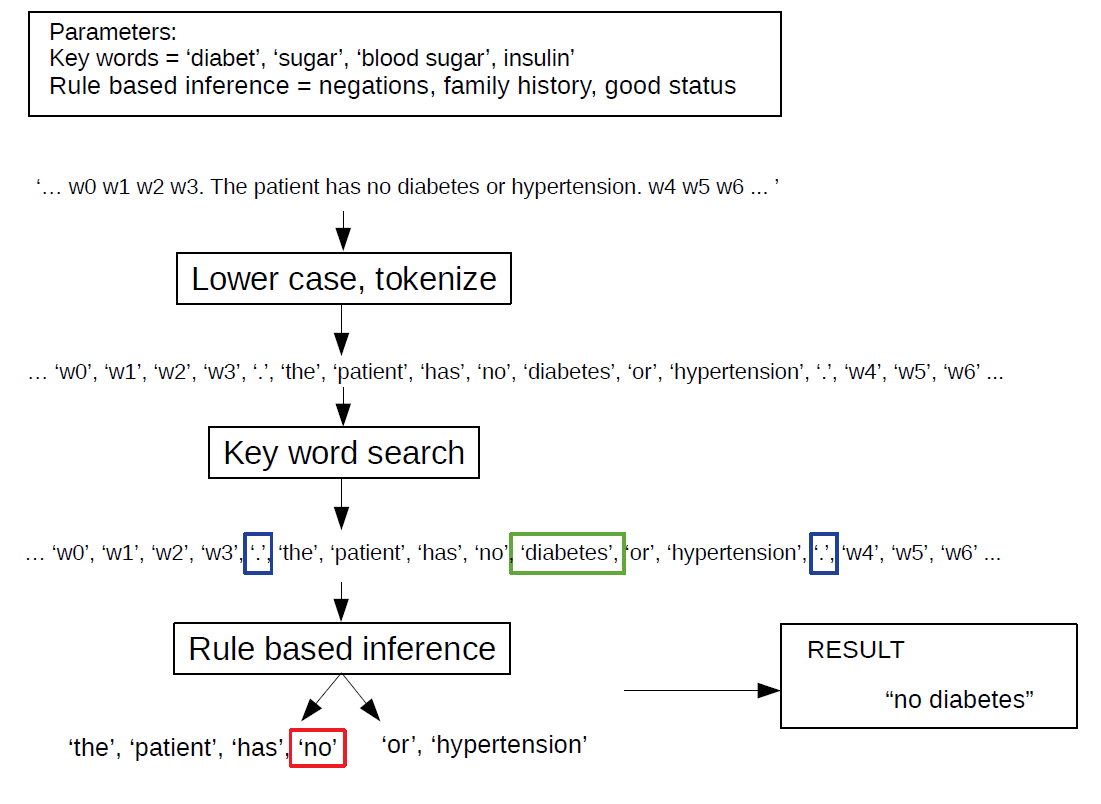  (a) |
| --- |
| 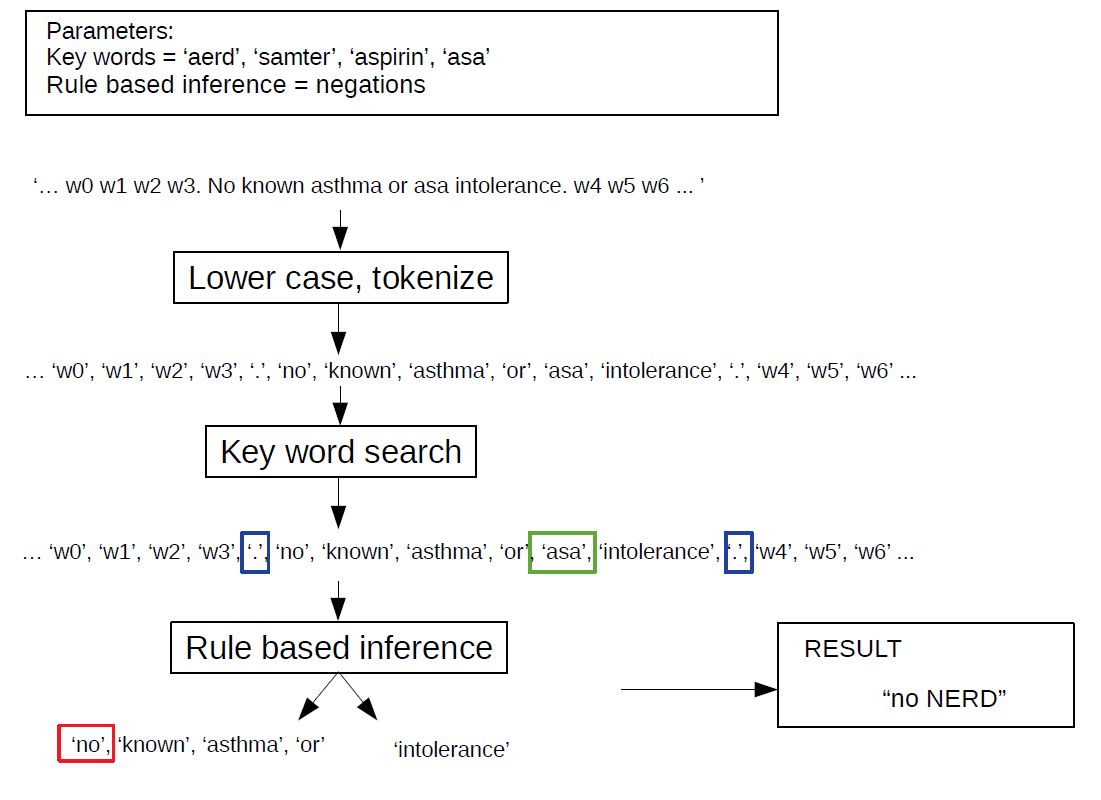  (b) |
| **Figure E1:** Examples of keyword search process of diabetes (a) and NERD (b) from clinical text. The keywords of the diabetes (translated to english) were: ‘diabetes’, ’sugar’, ’blood sugar’ and ’insulin’. The keywords for NERD were: ‘aerd’, ‘samter’, ‘aspirin’ and ‘asa’. In the information extraction process, the input text is first lower cased and tokenized. The tokens from the clinical text are compared to the keywords. When the clinical text token and the keyword match (green squares), the tokens before and after the matched keyword are fed for the rule-based inference. The principle is that the selected tokens should belong in the same sentence (separated by blue squares) and the distance from the matched keyword should be smaller than 10 tokens. The rule-based inference uses disease specific dictionaries (Table E1) for evaluating when matched keywords relate to the negation of the disease, family history or good medical status. |

**Table E2:** ICD-10 codes that were used for identifying chronic diseases.

| **Disease** | **ICD-10 codes** |
| --- | --- |
| Allergy | J30, J45.0 |
| Asthma | J45 |
| Cancer | C0-C97 |
| Cardiovascular diseases | I00-I99 |
| Chronic otitis media | H65, H66 |
| Chronic respiratory diseases | J40-J47, J80-J84, J90-94, G47.3 |
| Diabetes | E10-E14.8 |
| Gastroesophageal reflux | K21, R12, K44, K22.7 |
| Immunodeficiency | B20, D80-D84 |
| Mental disorders | F00-F99 |
| Memory disorders | F00-F02, G30-, F11-F19 |
| Mouth breathing | R06.5 |
| Musculoskeletal diseases | M00-M99 |
| Obesity | E65-E67 |
| Obstructive sleep apnea | G47.3 |
| Tonsils diseases | J03, J35 |

**Table E3:** Characteristics of the all patients and with the J30, J31, J32, J33 or J01 diagnosis. ENT = Ear nose throat diseases; NERD = Patient-reported non-steroidal anti-inflammatory drug -exacerbated respiratory disease; COM = Chronic otitis media; Rhinitis/rhinosinusitis = AR, NAR, ARS or CRS; Other diaseases ≠ AR, NAR, ARS, CRS; NAR = Nonallergic rhinitis; SD = Standard deviation

| **Variables** | **All patients** | **J30** | **J31** | **J32** | **J33** | **J01** |
| --- | --- | --- | --- | --- | --- | --- |
| Patients, n (%) | 5080 (100.0) | 1704 (33.54) | 1395 (27.46) | 1049 (20.65) | 554 (10.91) | 1379 (27.15) |
| Female, n (%) | 2848 (56.1) | 879 (51.6) | 782 (56.1) | 723 (68.9) | 219 (39.5) | 887 (64.3) |
| Age, mean (±SD) | 33.6 (20.7) | 20.2 (17.5) | 38.3 (19.1) | 40.2 (17.3) | 47.0 (15.9) | 37.0 (19.4) |
| Total number of visits*, all patients | 25927 | 4759 | 9896 | 8746 | 5651 | 8320 |
| Number of visits*, mean (±SD) | 5.1 (8.8) | 2.8 (6.2) | 7.1 (10.8) | 8.3 (10.3) | 10.2 (14.3) | 6.0 (11.3) |
| Number of visits*, Pulmonology and allergy, mean (±SD) | 0.1 (1.0) | 0.1 (0.6) | 0.1 (0.7) | 0.1 (1.2) | 0.1 (1.1) | 0.2 (1.8) |
| Number of visits*, Pulmonology, mean (±SD) | 1.6 (5.3) | 1.3 (4.4) | 2.3 (6.3) | 2.0 (6.1) | 2.8 (7.2) | 1.8 (6.6) |
| Number of visits*, ENT, mean (±SD) | 3.4 (5.8) | 1.5 (3.9) | 4.8 (7.3) | 6.3 (7.1) | 7.3 (10.5) | 4.0 (7.1) |
| Time interval between visits (days), mean (±SD) | 227.1 (321.2) | 279.2 (375.2) | 235.8 (309.9) | 209.8 (274.7) | 196.6 (217.6) | 205.8 (341.2) |
| Frequency of visits (visits/year, from first to last visit), mean (±SD) | 10.2 (35.6) | 4.2 (22.9) | 6.6 (19.5) | 8.5 (18.4) | 5.3 (7.6) | 22.4 (58.1) |
| Frequency of visits (visits/year, from first visit to end), mean (±SD) | 0.7 (1.2) | 0.4 (0.9) | 0.9 (1.3) | 1.1 (1.5) | 1.3 (1.6) | 0.8 (1.4) |
| Follow up time (days), mean (±SD) | 3103 (1257) | 3057 (1260) | 3126 (1210) | 3111 (1278) | 3175 (1267) | 3245 (1226) |
| Diabetes, n (%) | 535 (10.53) | 173 (10.15) | 171 (12.26) | 113 (10.77) | 79 (14.26) | 141 (10.22) |
| Chronic respiratory diseases, n (%) | 1957 (38.52) | 972 (57.04) | 565 (40.5) | 334 (31.84) | 211 (38.09) | 353 (25.6) |
| Obesity, n (%) | 510 (10.04) | 136 (7.98) | 212 (15.2) | 111 (10.58) | 62 (11.19) | 116 (8.41) |
| Mental disorders, n (%) | 960 (18.9) | 344 (20.19) | 271 (19.43) | 169 (16.11) | 71 (12.82) | 306 (22.19) |
| Memory disorders, n (%) | 129 (2.54) | 20 (1.17) | 53 (3.8) | 36 (3.43) | 22 (3.97) | 41 (2.97) |
| Cardiovascular diseases, n (%) | 1815 (35.73) | 512 (30.05) | 557 (39.93) | 421 (40.13) | 229 (41.34) | 519 (37.64) |
| Cancer, n (%) | 513 (10.1) | 63 (3.7) | 171 (12.26) | 123 (11.73) | 94 (16.97) | 198 (14.36) |
| Musculoskeletal diseases, n (%) | 1950 (38.39) | 443 (26.0) | 622 (44.59) | 509 (48.52) | 215 (38.81) | 668 (48.44) |
| Allergy, n (%) | 2590 (50.98) | 1704 (100.0) | 641 (45.95) | 378 (36.03) | 204 (36.82) | 310 (22.48) |
| Asthma, n (%) | 2257 (44.43) | 1256 (73.71) | 529 (37.92) | 371 (35.37) | 269 (48.56) | 381 (27.63) |
| NERD, n (%) | 197 (3.88) | 39 (2.29) | 48 (3.44) | 44 (4.19) | 98 (17.69) | 48 (3.48) |
| Immunodeficiency, n (%) | 25 (0.49) | 3 (0.18) | 7 (0.5) | 15 (1.43) | 1 (0.18) | 11 (0.8) |
| Immunodeficiency or its suspicion, n (%) | 114 (2.24) | 25 (1.47) | 34 (2.44) | 56 (5.34) | 9 (1.62) | 52 (3.77) |
| Obstr sleep apnea, n (%) | 480 (9.45) | 84 (4.93) | 272 (19.5) | 95 (9.06) | 76 (13.72) | 81 (5.87) |
| Mouth breathing, n (%) | 345 (6.79) | 92 (5.4) | 188 (13.48) | 65 (6.2) | 33 (5.96) | 42 (3.05) |
| Gastroesophageal reflux, n (%) | 280 (5.51) | 58 (3.4) | 112 (8.03) | 82 (7.82) | 31 (5.6) | 79 (5.73) |
| COM, n (%) | 314 (6.18) | 138 (8.1) | 72 (5.16) | 44 (4.19) | 16 (2.89) | 99 (7.18) |
| Tonsils diseases, n (%) | 323 (6.36) | 130 (7.63) | 86 (6.16) | 79 (7.53) | 18 (3.25) | 97 (7.03) |
| Rhinitis/rhinosinusitis, ≥ 2, n (%) | 859 (16.91) | 301 (17.66) | 490 (35.13) | 485 (46.23) | 173 (31.23) | 411 (29.8) |
| Other diseases, 0 diseases, n (%) | 529 (10.41) | 0 (0.0) | 128 (9.18) | 122 (11.63) | 61 (11.01) | 263 (19.07) |
| Other diseases, 1 disease, n (%) | 913 (17.97) | 181 (10.62) | 264 (18.92) | 222 (21.16) | 100 (18.05) | 288 (20.88) |
| Other diseases, 2 diseases, n (%) | 894 (17.6) | 261 (15.32) | 220 (15.77) | 203 (19.35) | 113 (20.4) | 246 (17.84) |
| Other diseases, 3 diseases, n (%) | 864 (17.01) | 441 (25.88) | 191 (13.69) | 133 (12.68) | 65 (11.73) | 169 (12.26) |
| Other diseases, ≥ 4 diseases, n (%) | 1880 (37.01) | 821 (48.18) | 592 (42.44) | 369 (35.18) | 215 (38.81) | 413 (29.95) |
| Number of any diseases, 1 disease, n (%) | 486 (9.57) | 0 (0.0) | 109 (7.81) | 86 (8.2) | 56 (10.11) | 235 (17.04) |
| Number of any diseases, 2 diseases, n (%) | 824 (16.22) | 155 (9.1) | 208 (14.91) | 167 (15.92) | 86 (15.52) | 249 (18.06) |
| Number of any diseases, 3 diseases, n (%) | 876 (17.24) | 246 (14.44) | 215 (15.41) | 204 (19.45) | 98 (17.69) | 236 (17.11) |
| Number of any diseases, 4 diseases, n (%) | 889 (17.5) | 417 (24.47) | 205 (14.7) | 152 (14.49) | 81 (14.62) | 184 (13.34) |
| Number of any diseases, ≥ 5 diseases, n (%) | 2005 (39.47) | 886 (52.0) | 658 (47.17) | 440 (41.94) | 233 (42.06) | 475 (34.45) |
| *during the whole follow up time | | | | | | |

**Table E4:** Cross-tabulation of J30, J31, J32, J33 and J01 patients (number of patients)

|  | J30 | J31 | J32 | J33 | J01 |
| --- | --- | --- | --- | --- | --- |
| J30 | 1704 | 176 | 87 | 50 | 73 |
| J31 | 176 | 1395 | 232 | 90 | 115 |
| J32 | 87 | 232 | 1049 | 0 | 260 |
| J33 | 50 | 90 | 0 | 554 | 72 |
| J01 | 73 | 115 | 260 | 72 | 1379 |

**Table E5:** Cross-tabulation of any CRS, CRSsNP, CRSwNP, ARS, RARS, any CRS AE and CRSwNP AE patients (number of patients). CRS = Chronic rhinosinusitis; CRSsNP = CRS without nasal polyps; CRSwNP = CRS with nasal polyps; ARS = Acute purulent rhinosinusitis; RARS = Recurrent ARS; CRS AE = CRS Acute exacerbation

|  | any CRS | CRSsNP | CRSwNP | ARS | RARS | any CRS AE | CRSwNP AE |
| --- | --- | --- | --- | --- | --- | --- | --- |
| any CRS | 1603 | 907 | 554 | 42 | 25 | 260 | 72 |
| CRSsNP | 907 | 907 | 0 | 31 | 18 | 223 | 0 |
| CRSwNP | 554 | 0 | 554 | 10 | 6 | 0 | 72 |
| ARS | 42 | 31 | 10 | 759 | 0 | 32 | 10 |
| RARS | 25 | 18 | 6 | 0 | 179 | 19 | 6 |
| CRS AE | 260 | 223 | 0 | 32 | 19 | 260 | 0 |
| CRSwNP AE | 72 | 0 | 72 | 10 | 6 | 0 | 72 |

| 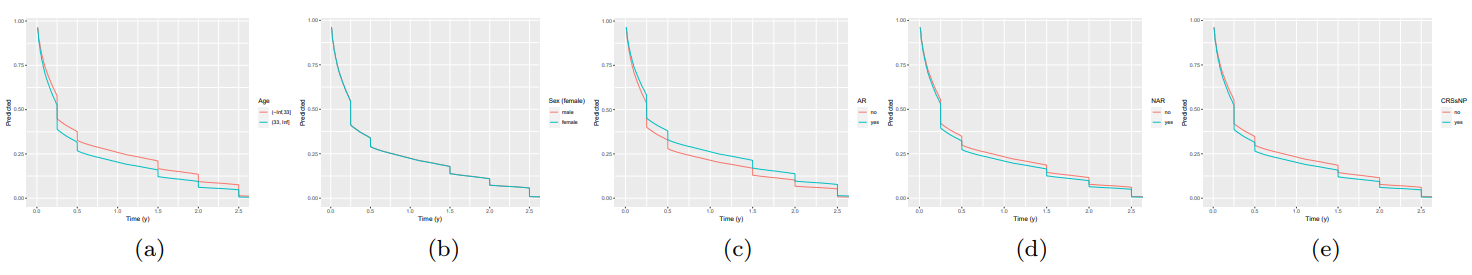  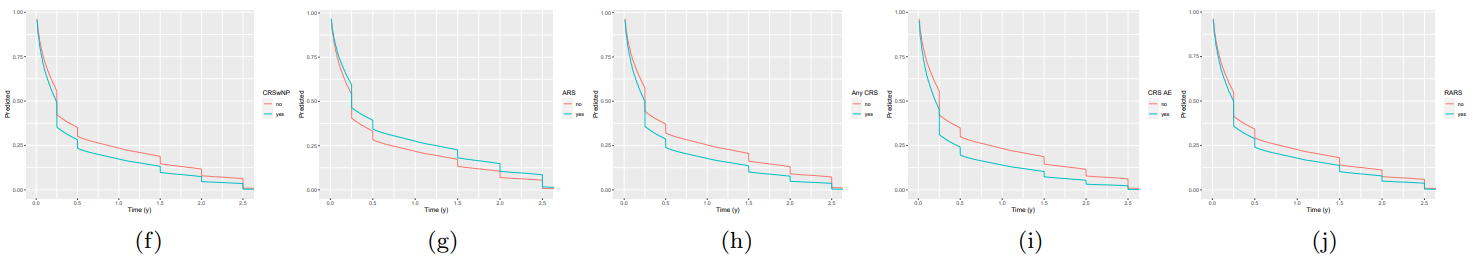  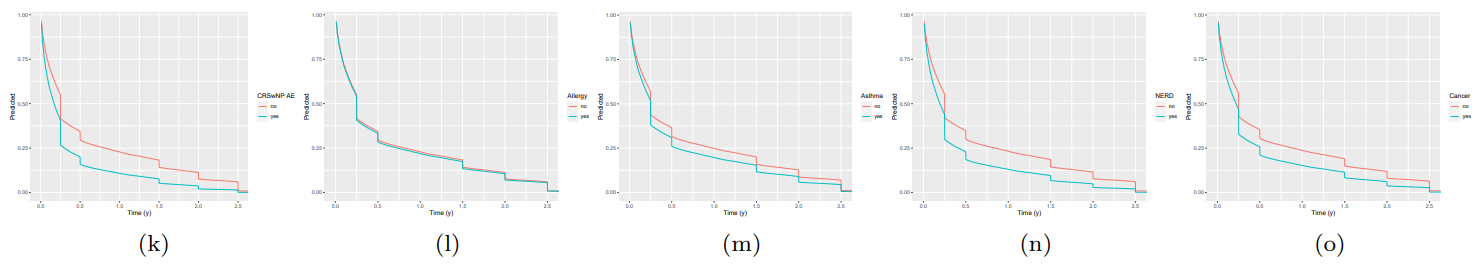  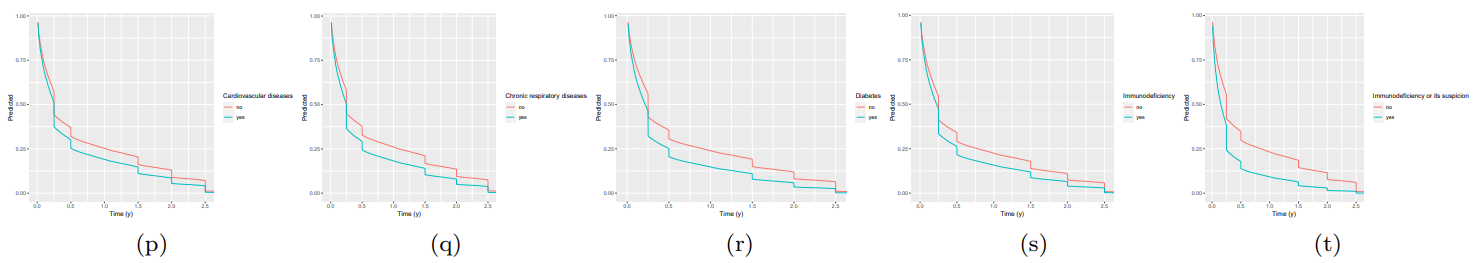  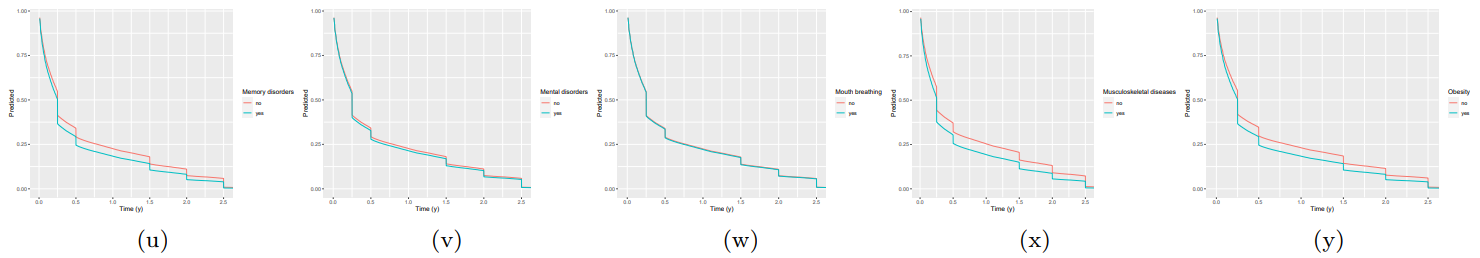 |
| --- |
|  |
| **Figure E2:** Survival curves illustrate time to the next visit for different variables. (a) Age > 33 years, (b) Sex (female), (c) AR = Allergic rhinitis, (d) NAR = Nonallergic rhinitis, (e) CRSsNP = CRS without nasal polyps, (f) CRSwNP = CRS with nasal polyps, (g) ARS = Acute purulent rhinosinusitis, (h) Any CRS, (i) CRS AE = CRS Acute exacerbation, (j) RARS = Recurrent ARS, (k) CRSwNP AE = CRSwNP Acute exacerbation, (l) Allergy, (m) Asthma, (n) NERD = Patient-reported non-steroidal anti-inflammatory drug -exacerbated respiratory disease, (o) Cancer, (p) Cardiovascular diseases, (q) Chronic respiratory diseases, (r) Diabetes, (s) Immunodeficiency, (t) Immunodeficiency or its suspicion, (u) Memory disorders, (v) Mental disorders, (v) Mouth breathing, (x) Musculoskeletal diseases, (y) Obesity. |
